# First cross-border outbreak of foodborne botulism in the European Union associated with the consumption of commercial dried roach (*Rutilus rutilus*)

**DOI:** 10.3389/fpubh.2022.1039770

**Published:** 2023-01-04

**Authors:** David Hendrickx, Carmen Varela Martínez, Matthias Contzen, Christiane Wagner-Wiening, Karl-Heinz Janke, Pablo Hernando Jiménez, Susanne Massing, Jeanette Pichler, Petra Tichaczek-Dischinger, Florian Burckhardt, Klaus Stark, Katharina Katz, Annette Jurke, Sebastian Thole, Rosa Carbó, Mariam Pascual del Pobil Ferré, Milagros Nieto, María Jesús Zamora, Ana Sisó, Pilar Pallares García, Sylvia Valdezate, Lars Schaade, Sylvia Worbs, Brigitte Gertrud Dorner, Christina Frank, Martin Bernhard Dorner

**Affiliations:** ^1^Landesgesundheitsamt Baden-Württemberg, Ministerium für Soziales, Gesundheit und Integration Baden-Württemberg, Stuttgart, Germany; ^2^European Centre for Disease Prevention and Control, European Programme for Intervention Epidemiology Training (EPIET), Stockholm, Sweden; ^3^National Centre of Epidemiology, Institute of Health Carlos III, Madrid, Spain; ^4^CIBER Epidemiología y Salud Pública (CIBERESP), Institute of Health Carlos III, Madrid, Spain; ^5^Chemisches und Veterinäruntersuchungsamt Stuttgart, Ministerium für Ernährung, Ländlichen Raum und Verbraucherschutz Baden-Württemberg, Fellbach, Germany; ^6^Chemisches und Veterinäruntersuchungsamt Karlsruhe, Ministerium für Ernährung, Ländlichen Raum und Verbraucherschutz Baden Württemberg, Karlsruhe, Germany; ^7^Landratsamt Böblingen, Veterinärdienst und Lebensmittelüberwachung, Böblingen, Germany; ^8^Gesundheitsamt, Landeshauptstadt Stuttgart, Stuttgart, Germany; ^9^Landesuntersuchungsamt Rheinland-Pfalz, Rhineland-Palatinate, Koblenz, Germany; ^10^Department for Infectious Disease Epidemiology, Robert Koch Institute, Berlin, Germany; ^11^GE 2.3 Epidemiologie übertragbarer Krankheiten, Landesamt für Gesundheit und Lebensmittelsicherheit, Oberschleißheim, Germany; ^12^Landeszentrum Gesundheit Nordrhein-Westfalen, North Rhine-Westphalia, Bochum, Germany; ^13^Servicio de Vigilancia y Control Epidemiológico, Dirección General de Salud Pública, Valencia, Spain; ^14^National Food Centre (CNA), Spanish Agency for Consumer Affairs Food Safety (AESAN), Majadahonda, Spain; ^15^National Centre of Microbiology, Institute of Health Carlos III, Madrid, Spain; ^16^Consultant Laboratory for Neurotoxin-Producing Clostridia (Botulism, Tetanus), Robert Koch Institute, Berlin, Germany

**Keywords:** foodborne botulism, fish, *Rutilus rutilus*, *Clostridium botulinum* type E, commercial dried roach, outbreak investigation

## Abstract

Botulism outbreaks due to commercial products are extremely rare in the European Union. Here we report on the first international outbreak of foodborne botulism caused by commercial salt-cured, dried roach (*Rutilus rutilus*). Between November and December 2016, an outbreak of six foodborne botulism type E cases from five unrelated households was documented in Germany and Spain. The outbreak involved persons of Russian and Kazakh backgrounds, all consumed unheated salt-cured, dried roach—a snack particularly favored in Easter-European countries. The implicated food batches had been distributed by an international wholesaler and were recalled from Europe-wide outlets of a supermarket chain and other independent retailers. Of interest, and very unlike to other foodborne disease outbreaks which usually involves a single strain or virus variant, different *Clostridium botulinum* strains and toxin variants could be identified even from a single patient's sample. Foodborne botulism is a rare but potentially life-threatening disease and almost exclusively involves home-made or artisan products and thus, outbreaks are limited to individual or few cases. As a consequence, international outbreaks are the absolute exception and this is the first one within the European Union. Additional cases were likely prevented by a broad product recall, underscoring the importance of timely public health action. Challenges and difficulties on the diagnostic and epidemiological level encountered in the outbreak are highlighted.

## 1. Introduction

Botulism is a rare but life-threatening paralytic illness in humans and animals resulting from the potent botulinum neurotoxins (BoNTs) produced by seven distinct anaerobic bacteria species of the genus *Clostridium* (*C. botulinum* Groups I–IV, *C. baratii, C. butyricum*, and *C. sporogenes*) whose spores are found widespread in soil and marine sediments (1). The botulinum neurotoxins (BoNTs), which are the most lethal substances known to mankind, are subdivided into eight serotypes (A to H) out of which types A, B, E, and rarely F are associated with foodborne botulism in humans (2, 3).

On the molecular level BoNTs cleave the SNARE proteins, essential components for the fusion of neurotransmitter-loaded vesicles with the presynaptic membrane. Here, each serotype cleaves at a unique position in one of the SNARE proteins SNAP-25, VAMP or syntaxin which prevents fusion of neurotransmitter-loaded vesicles with the synaptic cell membrane. The resulting block in neurotransmitter release leads to paralysis of the inverted muscle which is the hallmark of botulism. Foodborne botulism, one of three disease forms besides infant and wound botulism, is an intoxication caused by the ingestion of botulinum neurotoxins preformed in foods. Paralysis occurs usually within 12–72 h of ingestion. Early indications of intoxication often include blurred vision, difficulty in speaking and swallowing, weakness and fatigue, followed by flaccid and respiratory paralysis in severe cases, which require mechanical ventilation. Together with the timely administration of antitoxin intensive supportive care is the main mode of treatment (2).

Botulism is rare in the European Union (EU), with 80–132 cases reported annually to the European Center for Disease Prevention and Control (ECDC) between 2008 and 2021 (4). Like in many countries of continental Europe foodborne botulism accounts for the majority of botulism cases (5, 6). In Germany over 70% of the 154 total botulism cases notified between 2001 and 2020 were foodborne (7). Type A and B BoNTs are most frequently encountered and associated with various meat and vegetable products (5, 6, 8–10), whereas foodborne botulism due to serotype E is almost exclusively associated with aquatic products and more often seen in the northern hemisphere (11).

Foodborne botulism results from the consumption of food in which BoNT-producing *Clostridium* spp. where able to grow and produce toxins. This requires certain suitable conditions: (i) anaerobic conditions; (ii) pH-values >4.5; temperatures above 3°C; (iii) salt concentration lower than 5 % which vary somewhat between the involved species (12). Interestingly, growth occurs usually in foods in which the natural flora has been greatly reduced such as in canned, cured or fermented products; indicating that BoNT-producing clostridia have a low competition rate. Their spores show a high resistance against heat and chemicals and as a consequence modern food industry applies strict measures to assure their destruction or to prevent their germination or growth (12, 13) including challenge testing with toxic and non-toxic strains (14–17). As a consequence, modern commercial products are extremely rarely involved in foodborne botulism in particular within the EU, unlike home-made or artisan products which cause the majority of cases (18–20).

Here we describe the epidemiological and microbiological investigation of an outbreak due to commercial cured and dried roach (*Rutilus rutilus*) which was distributed across several countries within the EU. Four cases occurred in Germany and Spain within a week and were followed by two additional cases in Germany after the product had been recalled. A possible linked seventh case occurred in Germany about 6 months after the first illness.

## 2. Materials and methods

### 2.1. Outbreak case definition

A probable outbreak case was defined as a resident in EU/EEA with clinical symptoms compatible with botulism and disease onset on or after 1st of November 2016, and exposure to salted and dried roach. A confirmed outbreak case was defined as a probable case with detection of BoNT/E or a *bont/*E-coding gene in a clinical sample (21).

### 2.2. Epidemiological outbreak investigation

Health officers from the local health authorities interviewed patients and their family/household members as soon as possible after the onset of illness with a focus on recent food consumption history. Food samples from the households were collected whenever possible. Data on public health measures and trace-back investigations were collated from available Rapid Alert System for Food and Feed (RASFF) information. Relevant health and food authorities were contacted directly for further clarification as required.

### 2.3. Laboratory analysis

Suspected cases of botulism can be confirmed by showing the presence of BoNT in clinical samples such as serum, stool or gastric content. This is frequently done by the mouse bioassay (MBA) were a sample is injected intraperitoneally and the animal is observed for botulism symptoms. A sample resulting in botulism symptoms needs to be confirmed by neutralizing the BoNT(s) action by serotype-specific anti-toxins to exclude other deleterious substances (22). Other validated methods including ELISA, Endopep-MS or Endopep-ELISA may be used to detect the BoNT (23–25). Alternatively, clinical suspected botulism can be confirmed by the presence of BoNT-producing species from feces e.g., by PCR on the *bont* genes. In addition, the suspected food (left-overs) can be analyzed ideally for the toxin or alternatively for the producing organisms.

For the German cases, stool and serum samples were sent to the German national Consultant Laboratory for Neurotoxin-producing Clostridia (botulism, tetanus) (CL-NTC) located at the Robert Koch Institute (RKI). Serum was tested by mouse bioassays (MBA) for the presence of BoNTs, while PCR-based analyses for *bont* and *ntnh* (non-toxin non-hemagglutinin; a surrogate marker for BoNT-producing *Clostridium* species) genes were performed on the stool samples after anaerobic enrichment culture (26–28). Food samples were analyzed by the State Investigatory Office (LUA) in Koblenz (Rhineland-Palatinate), the Chemical and Veterinary Investigatory Offices (CVUA) in Stuttgart and Karlsruhe (both Baden-Württemberg), and the CL-NTC. Food was tested with and without prior enrichment culture for the presence of *bont* genes (26, 29). Toxin testing of food samples was done by MBA and sandwich ELISA (30).

Samples of the Spanish cases were initially analyzed by a regional lab, and afterwards by the National Center of Microbiology, Institute of Health Carlos III. Fish samples were analyzed by the National Food Center, Spanish Agency for Consumer Affairs Food Safety (AESAN). Serum and fish samples were tested by MBA for the presence of BoNTs.

Isotope analysis of roach samples was performed by a commercial laboratory (Isolab, Schweitenkirchen, Germany). Stable isotope δ^34^S signatures (ratio of two stable isotopes of Sulfur (^34^S/^32^S) in a sample against the equivalent ratio in a known reference standard) of fish samples collected during the outbreak investigations were compared with reference samples from the various fishing grounds of roach products implicated in the outbreak (31).

### 2.4. Molecular analysis of obtained isolates

Genomic DNA was purified and sequences of the 16S rDNA and the *bont* genes were obtained (32). Sequence reads were assembled in Geneious 9 (Biomatters Limited, Auckland, New Zealand) and compared with the NCBI GenBank database records using the built-in BLAST algorithm. The 16S sequence allows to identify the underlying species (20). For BoNT/E subtyping, amino acid sequences were aligned in Geneious and compared with the 12 known BoNT/E subtypes [E1–E12; (3)]. A dendrogram was calculated from the amino acid alignment by the built-in neighbor-joining method.

## 3. Results

### 3.1. Investigation of two initial foodborne botulism cases notified in Germany

**Figure 1:**
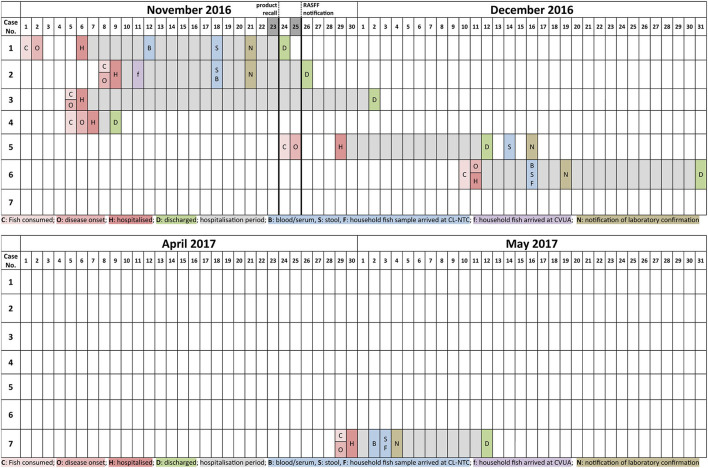
Timeline of outbreak events including information on the dates of fish consumption (C), disease onset (O), hospitalization (H), and discharge from hospital (D) as well as the dates were samples of blood/serum (B), stool (S), leftovers of household fish arrived at the CL-NTC (F) or CVUA (f). Furthermore, the date of mandatory notification of the laboratory results to the local health authorities (N) is indicated.

Cases 1 and 2, who experienced symptom onset on November the 2nd and 8th 2016, respectively (Table 1; Figure 1), were too unwell to provide information prior to their hospital admission. Family members indicated that case 1 had not consumed any home-canned goods within 48 h prior to symptom onset, but had eaten cold-smoked herring purchased at the fish counter of a local supermarket chain outlet specialized in Russian/Eastern European foods. On Nov 8, local food safety officers took several herring samples from the outlet for laboratory investigations, all of which were tested negative for the presence of BoNT toxin and *bont* genes.

**Table 1:** Summary of information on patients, symptoms, treatment, and laboratory results.

**Case no., country**	**Sex**	**Age (years)**	**Fish consumed**	**Disease onset**	**Admission date**	**Ventilary support**	**Antitoxin administration**	**Serum testing^a^**	**Stool testing^b^**	**Fish testing^a^^,^^b^**
1, DE^C^	M	40	01.11.2016	02.11.2016	06.11.2016	Yes	No	Negative	*bont*/E gene	n.a.
2, DE^C^	M	37	08.11.2016	08.11.2016	09.11.2016	Yes	Yes	BoNT/E	*bont*/E gene	*bont*/E gene
3, ES^P^	F	46	05.11.2016	05.11.2016	06.11.2016	Yes	Yes	Negative	Negative	BoNT/E + *bont*/E gene^$^
4, ES^P^	M	46	05.11.2016	06.11.2016	08.11.2016	No	No	Negative	Negative	BoNT/E + *bont*/E gene^$^
5, DE^C^	M	44	24.11.2016	25.11.2016	29.11.2016	No	No	n.a.	*bont*/E gene	n.a.
6, DE^C^	F	55	10.12.2016	11.12.2016	11.12.2016	Yes	No	BoNT/E	*bont*/E gene	BoNT/E + *bont*/E gene
7, DE^C^^,^^L^	M	54	29.04.2017	29.04.2017	30.04.2017	Yes	Yes	BoNT/E	*bont*/E gene	*bont*/E gene

DE, Germany; ES, Spain; n.a., not available.

C Confirmed case = consumed roach + clinical symptoms compatible with botulism + detection of BoNT/E or bont/E gene in patient sample.

P Probable case = consumed roach + clinical symptoms compatible with botulism.

L Possibly linked case.

a Mouse bioassay for BoNT/E toxin.

b PCR for bont/E toxin gene.

$ No household samples available. Fish samples were taken from the supermarket where cases had purchased roach.

Interviews with case 2's family members indicated that he had eaten salt-cured, dried roach (*Rutilus rutilus*) on the day of symptom onset, and that no other family members had eaten the roach. Several leftover food samples were collected from case 2's household for laboratory investigation: an opened jar of preserved sardines, a piece of smoked bacon, a leftover tail-end of the roach that had been partially consumed by case 2, and two dried roaches that had been labeled as eviscerated but in which remaining entrails were noted in the laboratory. While all case 2's household samples were tested negative for BoNT by MBA, enrichment cultures performed on samples taken from the leftover roach tail-end and one of the unconsumed dried roaches were found positive for *bont*/E by PCR.

Questioned after his recovery on Dec 7, case 1 also confirmed the consumption of salt-cured roach—which he described as uneviscerated—shortly prior to symptom onset. None remained for laboratory testing. An investigation into the source of the salt-cured roach that had been consumed by cases 1 and 2 showed that both had been purchased from two different outlets of the same supermarket chain. Consumption of salt-cured roach sold by this chain of stores was therefore considered as the source of intoxication.

### 3.2. Trace-back, identification, and recall of the implicated dried roach products

The implicated supermarket chain specializes in the retail of international food items with a focus on Russian/Eastern European specialties and operates in 15 European countries. Investigations showed that the supermarket chain sold several different salt-cured roach products, representing varying fishing grounds and distribution chains. All products were sourced from the same international wholesaler. Following the positive PCR tests for *bont*/E in the roach samples taken from case 2's household, the wholesaler communicated an urgent recall to all its European customers on Nov 23 for salt-cured, eviscerated roach originating from Lithuania. The recall was amended the following day to include eviscerated and uneviscerated roach products from The Netherlands. All affected retail stores in Germany and other EU-member states were notified of the recall and requested to remove the fish products from their stock and to inform their customers of the recall, which was done *via* in-store posters. The international wholesaler also issued a press release, which was rapidly picked up by the German-language press. As the press release was perceived not to have penetrated Germany's Russian-language media platforms, the RKI actively communicated the press release and its public health importance to several of them.

German authorities posted an alert on the European Epidemic Intelligence Information System for food- and waterborne diseases (EPIS-FWD; now: EpiPulse) on Nov 22, 2016, followed by a notification on the RASFF system on Nov 25, 2016, thereby formally notifying other EU countries of the two documented botulism cases and the details of the implicated roach products. Health and food safety authorities in affected EU member states conducted additional investigations in their respective countries to ensure that the recall was adequately implemented.

RASFF updates eventually referred to four distinct dried roach products targeted for recall, which were identified using product numbers assigned by the international wholesaler. Table 2 summarizes key characteristics of each product and its distribution chain up to the international wholesaler. As initial investigations were unable to ascertain whether the outbreak was related to eviscerated or uneviscerated roach, and given that the fish were sold individually, picked from unpacked wares in open counters, the decision was made to include all four products in the RASFF alert with due-by-dates ranging from Nov 06, 2016 to Mar 12, 2017.

**Table 2:** Product description, producers/distributors, origin and δ^34^S of the four roach products that were distributed to outlets in Europe by the international wholesaler.

**Product number**	Product labeled eviscerated^*^	**Main producer/distributor^§^**	**Origin of fish^#^**	**Reference δ^34^S value**
1	No	Company A, The Netherlands	Ijsselmeer, The Netherlands	7.8
2	Yes	Company A, The Netherlands	Ijsselmeer, The Netherlands	7.9
3	Yes	Company B, Lithuania	Szczecin Lagoon, Poland	11.5
4	Yes	Company C, Lithuania	Curonian Split, Lithuania	3.7

n.a., not available.

* Indicates whether the roach product was labeled and sold as an eviscerated or uneviscerated product; source: RASFF 2016.1621.

§ Describes producers/distributors that delivered the roach product directly or indirectly to the international wholesaler; source: RASFF 2016.1621.

# Describes the fishing grounds from where the roach product originated; source: RASFF 2016.1621.

### 3.3. Linked botulism cases in Spain and new cases in Germany

Following the publication of the EPIS-FWD and RASFF notifications, Spanish health authorities indicated that two additional cases of human foodborne botulism had been reported in Spain (cases 3 and 4; Table 1; Figure 1). The two cases, a couple with a Russian background, reported the consumption of salt-cured roach (described as uneviscerated) on Nov 5, 2016 and fell ill within the following 24 h. While the man only experienced gastroenteric illness (although electromyography showed abnormalities consistent with peripheral nerve impairment due to botulism), the woman developed the full paralytic clinical picture of foodborne botulism, required ventilatory support. Serum and fecal samples from both Spanish cases were tested negative for BoNT by MBA. Additional samples from case 3 were tested negative for *bont*/E by PCR. The couple had purchased the fish from a local independent retailer that had been supplied by the same international wholesaler that was implicated in the German cases, who in turn received the roach products directly or indirectly from Dutch and Lithuanian producers/distributors (Table 2). Fish samples presumed to come from the same batch as the fish consumed by the two Spanish cases were obtained from the retailer and tested positive for BoNT by MBA. The bulk box label in which the roach had been delivered to the supermarket indicated that it originated from a Dutch producer.

Notwithstanding the initiated control efforts in Germany, two additional cases of foodborne botulism were notified in Germany following the EU-wide recall notice (Table 1; Figure 1). Case 5, a male of Russian background and residing in the German Free state of Bavaria, fell ill on Nov 25, 2016—two days after the recall—following the consumption of salt-cured roach on the 24th purchased from one of the implicated supermarket outlets prior to the recall. While an initial laboratory analysis of case 5's serum remained inconclusive, a fecal sample sent to the CL-NTC revealed the presence of *C. botulinum* type E.

A 6th case of botulism was hospitalized in the German state of North Rhine-Westphalia on Dec 10, 2016, a female patient of Kazakh background. She and her husband had both consumed salt-cured roach (described as uneviscerated) purchased prior to the recall in an outlet of the implicated supermarket chain. While the woman developed fulminant botulism, her husband experienced no symptoms. Stool and serum samples of case 6 and of her husband, as well as leftover material of the consumed roach were sent to the CL-NTC for laboratory testing. BoNT/E was detected from the woman's serum by MBA. Her stool also tested positive for *bont*/E by PCR. No *bont*/E signal was obtained from the husband's stool sample. Leftover material of the partially consumed roach was found positive for BoNT/E and *bont*/E.

A potentially linked 7th case occurred on Apr 29, 2017 in the German state of Lower Saxony involving a male patient of Russian ethnicity (Figure 1). Here, dried roach was obtained from another supermarket chain also specialized in Russian/Eastern European food. Although this supermarket chain procures its roach products from a different wholesaler than the one implicated in cases 1 to 6, said wholesaler also did source them indirectly from Lithuanian producer/distributor B involved in the former cases. There was no information available whether the fish was eviscerated or not.

### 3.4. Further comparison of the outbreak isolates

**Figure 2:**
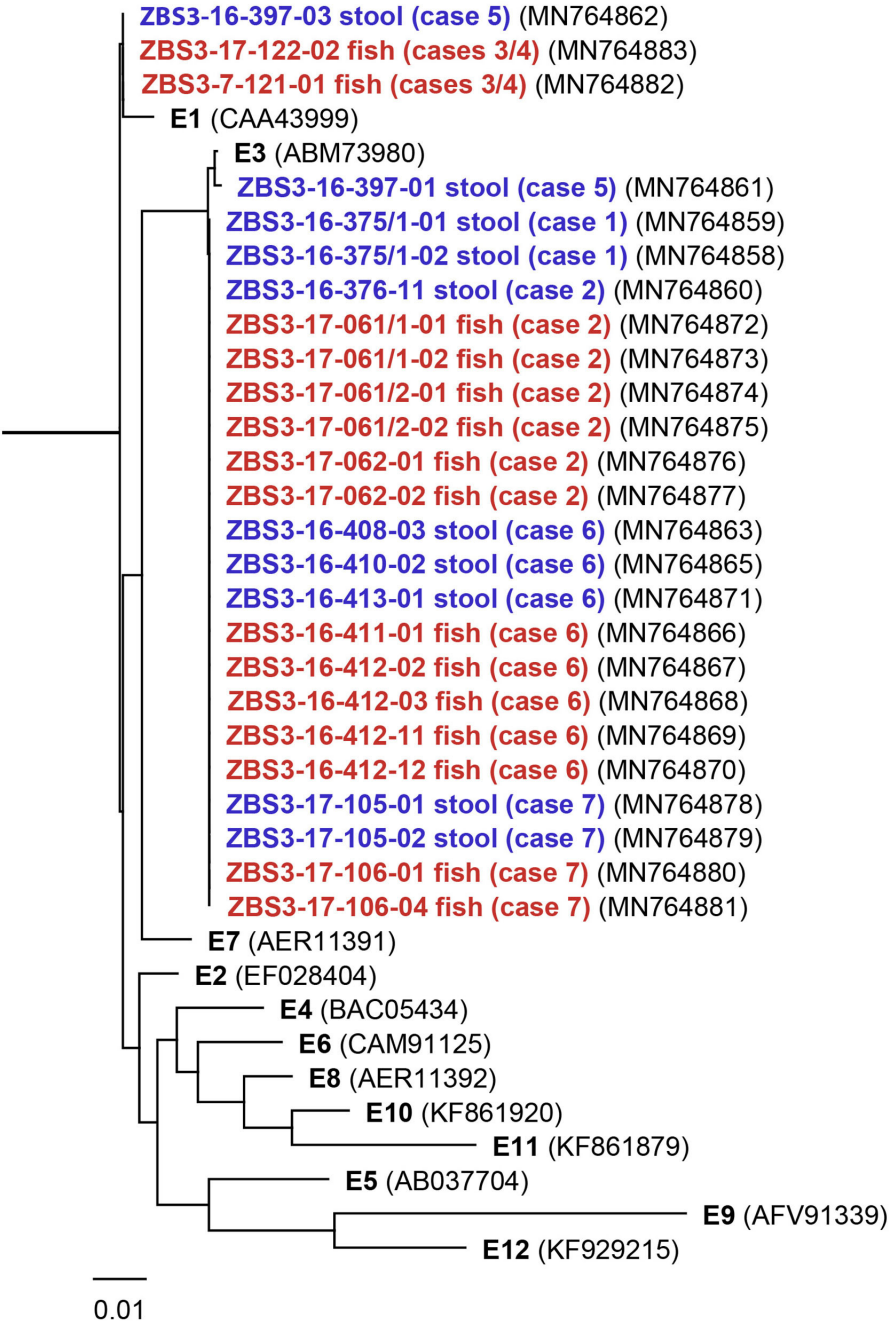
Dendrogram of the translational alignment of the 12 described prototypic BoNT/E subtypes (E1 to E12; black) compared to the obtained BoNT/E subtype amino acid sequences from implicated stool (blue) or fish (red) samples. The scale bar indicates amino acid substitutions per site. GenBank accession numbers are given in parentheses.

The analysis of the 16S rDNA sequences revealed that all isolates belong to *C. botulinum* Group II species. This species accounts for the vast majority of type E producing strains and covers subtypes BoNT/E1, E2, E3, and E6 to E12. Whereas, subtypes E4 and E5 are produced by *C. butyricum* and are only very seldom involved in foodborne outbreaks (1, 3). It has been observed that certain BoNT subtypes are restricted to single regions/countries while other show a broader distribution (33, 34). To obtain potential information on the origin of the fish we sequenced the *bont*/E genes of all isolates to elucidate the underlying BoNT/E subtype(s) based on their amino acid sequences. BoNT/E sequences of 25 isolates obtained by the CL-NTC were compared to the twelve known BoNT/E subtypes (3). Figure 2 depicts a dendrogram based on the alignment of the amino acid sequences of the known 12 subtypes and the 25 isolates from the 6 outbreak cases as well as the potential linked 7^th^ case. All but two *bont*/E sequences from the German cases (cases 1, 2, 5, 6, and 7; fish or feces) were identical and of the E3 subtype. The stool sample of case 5 returned two isolates, one encoding BoNT/E1 (16-397-03), the other encoding BoNT/E3 (16-397-01) which contains 3 nucleotide substitution compared to all other E3 isolates resulting in the change of three amino acids. Noteworthy, the BoNT/E3 isolate 16-379-01 is more closely related to the prototype E3 isolate Alaska E43 (GenBank: ABM73980). The isolates obtained from the Spanish fish samples (17-121-01, 17-122-02; cases 3/4) belong to the E1 subtype and have an identical *bont*/E sequence as the one isolate from Bavaria (16-397-03).

### 3.5. Analysis of fish from recalled batches

Three boxes of recalled roach including eviscerated fish from producer/distributor A and uneviscerated fish from producer/distributor C were available for testing and analyzed for the presence of BoNT-producing *Clostridium* species. In total 148 fish ranging in weight from 116.6 to 298.8 g (mean: 184.1 g) were subjected to anaerobic enrichment culture and tested for the presence of *bont*/E (26) and *ntnh* genes (27). None delivered any positive results. We did note however, that while two boxes were supposed to contain eviscerated fish only, three out of 100 fishes were found uneviscerated and several not fully eviscerated.

### 3.6. Isotope analysis: Tracing the origin of the affected roaches

The supermarket chain sold dried roaches obtained from three distinct producers (Table 2) from The Netherlands (North Sea), Poland and Lithuania (both Baltic Sea), as loose fish to be individually wrapped and purchased. To obtain more information on the potential origin of fish, isotope analysis (stable isotope δ^34^S signatures) was performed on single samples from all three potential suppliers and compared to the δ^34^S values from the roach samples of the cases' households. As the values for the roach samples showed quite a degree of variation, no clear attribution of the cases' roach samples to a single fishing ground was possible (data not shown).

## 4. Discussion

The conducted investigations identified salt-cured, dried roach as the vehicle of BoNT/E intoxication in four German (laboratory confirmed) and two Spanish (laboratory unconfirmed) cases of foodborne botulism in November and December 2016. The four German cases were from different households and had all purchased salt-cured roach in separate outlets of the same Russian/Eastern European specialty food supermarket chain, which sourced its roach products from an international wholesaler. The two Spanish cases were a couple who had purchased roach in a local independent supermarket in Spain, which was also stocked by the same international wholesaler. All cases recovered after 3–26 days of hospitalization and had all a background in Russia or Kazakhstan. In Eastern European cultures, salt-cured roach is often consumed without further preparation as a snack or side dish. The presence of the *bont*/E gene or BoNT/E toxin was confirmed in roach samples taken from two German outbreak households, as well as samples taken from the implicated Spanish supermarket. Trace-back investigations identified the European wholesaler of the concerned roach products, prompting a successful broad EU-wide recall. The described outbreak, which affected persons of Russian/Kazakh ethnicity, and in which two cases were apparently not reached by the recall, highlights the importance of the application of target-group specific outbreak communication strategies, especially in regard to language barriers.

To our knowledge the outbreak reported here represents the first cross-national outbreak of foodborne botulism observed in the European Union involving a commercial product. In addition, it constitutes the first foodborne botulism outbreak involving different federal states in Germany since the introduction of the mandatory reporting of botulism in 2001. Even if commercial food products are distributed to several countries, outbreaks of botulism in the EU are usually limited to a single country and individual cases (19, 28, 35, 36). Even worldwide we could only identify two cross-national outbreaks (37, 38). Notably, one involved salt-cured, uneviscerated whole fish with cases in Israel and the US (38).

Food traceability proved a major challenge in the frame of this outbreak. The supermarket chain implicated in the German cases sold four distinct salt-cured roach products, constituting three different fishing grounds and distribution chains (Table 2). While the roach products were shipped in labeled boxes, they were unpacked and sold as individual items in open fish counters at supermarket outlets. Sold fish were bagged and labeled with a product description that did not provide a traceable article number or information on the origin of the fish. As a result, it proved impossible to identify exactly which of the four salt-cured, dried roach products was the source of the outbreak. Therefore, given the urgency in avoiding possible additional cases of foodborne botulism, a decision was made to issue a recall for all four products (Table 2) within a 4-month due-by-date range. The ensuing communication over RASFF brought further complications to light, including product mislabeling, unclear distribution chains, and uncertainty regarding the fishing grounds/producers where the various roach products originated from. The implementation of a more stringent food traceability standard for unpacked, individually sold fish products would certainly facilitate trace-back investigations in possible future outbreaks. The inconclusive findings regarding the origin and distribution chain of the affected fish has precluded a targeted investigation into possible production factors that might have contributed to bacteria growth and toxin formation.

A later attempt to attribute the source of the affected roach by isotope analysis proved unsuccessful. The observed δ^34^S signatures varied greatly, suggesting that the fish were taken from different fishing grounds, or that signatures of individual fish could vary significantly even within the same fishing ground. It may well be possible that a batch of dried roach accumulates fish from different netting areas (estuarine vs. further offshore) and as such of varying isotope signatures which may conceal the attribution of samples to a certain producer on the basis of an isotope analysis. The observed heterogeneity in δ^34^S signatures amongst outbreak fish samples might also indicate that fish from multiple origins was involved and the outbreak is the consequence of a mere temporal coincidence of individual cases. However, when considering the high number of dried roaches that are sold every year within the EU without any associated botulism cases, it seems plausible that the outbreak originates from mishandling of the fresh fish (e.g., temperature abuse) or a faulty production or distribution process which might resulted in ungutted or not fully eviscerated fish bodies with a higher risk of spore contamination. Fish, in particular dried or cured fish products which are consumed unheated, are from time-to-time associated with botulism (39–43). Noteworthy, foodborne botulism involving (commercial) dried fish products is more frequently observed in Eastern European countries from outside the EU (44, 45).

As certain BoNT subtypes can be linked to restricted geographic location we performed subtyping of the BoNT/E sequence (33, 34, 46). Subtyping was conducted on the 21 isolates from the 6 cases and 4 isolates from the possibly linked case 7. Two subtypes (E1 and E3) were identified (Figure 2). Whereas, the two Spanish fish isolates (cases 3 and 4) and one isolate from stool from case 5 belonged to the E1 subtype, all others belonged to the E3 subtype. Remarkably, the stool sample from case 5 delivered two subtypes E1 and E3, with the E3 subtype having 3 amino acid substitutions compared to all the other E3 isolates. Unfortunately, both E1 and E3 are very frequently encountered by the CL-NTC and other European labs (9, 47), reflecting a broad distribution in the northern hemisphere, including North and Baltic Sea, and thereby precluding any attribution. The identification of different BoNT subtypes within a single outbreak is very unusual in human botulism but has been once reported for BoNT/E outbreaks among waterfowl (48), and results likely from fish contaminated by more than one strain.

Fish will naturally take up spores of *C. botulinum* by feed and spores of *C. botulinum* can be found in their intestine. In addition, contamination can occur externally e.g., by mud or soil particles (48, 49). Spores of *C. botulinum* type E have been found in soil sediments and fish from the Baltic and North Sea (50–52). The comparably high rate of seafood products involved in botulism (11, 44, 53) indicates the high natural risk for spore contamination in these products. Therefore, the germination of contaminating spores must be prevented to ensure product safety.

In dried, salt-cured fish, environmental conditions such as a water activity below 97%, NaCl concentrations above 5% and a pH below 5 usually prevent growth of *C. botulinum* Group II and thus production of the toxin (44). Nevertheless, when water activity, pH and/or NaCl concentration are not properly adjusted in a timely manner throughout the whole fish, spores might germinate and produce toxin even under refrigerated temperatures (12, 44, 54). Chilled storage can prolong the shelf-life (44) but dried fish is not always stored and sold under chilled conditions. Notably, the observed variation in the size of individual fishes, ranging from 116.6 to 298.8 g, and the inclusion of uneviscerated or not fully eviscerated fish (see above) calls for extra safety margins in the production and distribution process. In particular uneviscerated, salt-cured fish has been previously subject to food recalls/warnings (55–57) and has also been recurrently associated with foodborne botulism (38, 42, 43, 58). Since spores are often associated—however not exclusively—with the viscera, proper evisceration can decrease the risk of *C. botulinum* growth and toxin production, and as such reduce the risk of *C. botulinum* associated disease in products consumed unheated (54, 59). It would be advisable that fish products traditionally consumed unheated should be appropriately eviscerated and regularly monitored for the presence of BoNT-producing clostridia. Nonetheless, fish preservation by salting and drying has been applied successfully throughout the history of mankind and botulism is still a very rare incidence in an otherwise safe process. How exactly the contamination of fish with *C. botulinum* spores developed to botulism in this outbreak remains speculative. No insights in the production process at the various production sites were possible but it can be speculated that, in light of the good safety record of dried roach within the EU, the affected roach was either subjected to extended incubation at elevated temperatures prior to salting/drying, improperly produced (e.g., too short incubation time during salting or drying; fish not fully submerged in brine/salt), or underwent prolonged storage at elevated temperatures during distribution or before consumption, thereby providing the environmental conditions for spore germination and toxin production.

In essence, neither the food trace-back analysis nor the identified subtypes, nor the analysis of the δ^34^S signatures allowed us to attribute the outbreak to a certain fishing ground/producer. Nevertheless, the data does not exclude that flaws in the process of a single producer or trawler allowed for germination and toxin production in the products.

Of note in this outbreak of botulism was the diversity of the observed severity of symptoms. The Spanish married couple (cases 3 and 4) had consumed the same roach, yet case 4 (male) only developed gastrointestinal disturbances without severe neurological complications, while case 3 (female) developed the severe paralytic symptoms associated with botulism. Similarly, the husband of case 6 developed no illness regardless of having mentioned to have eaten the same roach as his spouse. This, and the fact that the leftover fish from case 2's household tested positive only for the *bont*/E gene but not for the toxin itself, indicates that the toxin is likely distributed non-homogeneously throughout a contaminated food. Indeed, Justinus Kerner already noted in his very first description of botulism in 1817 that the severity in symptoms could vary substantially from symptomless cases to rapid death even among persons who consumed of the same food (60); this peculiarity has been seen frequently but not exclusively for type E (61–68). In yet another recent case of type E3 foodborne botulism in Germany, caused by in The Netherlands self-caught and home-cured dried roach, husband and wife, both of Russian ethnicity, consumed the fish. Only the husband—who unlike his wife had consumed the fish's roe—developed severe symptoms. The most likely explanation is that the bacteria/toxin was not homogenously distributed throughout the fish and perhaps concentrated in the roe. Said heterogeneity could be attributed to non-homogenous conditions (e.g., in pH value, free water, salt concentrations, presence of other competing microorganisms) preventing spread, growth and toxin production in some parts of the food and favoring it in others. Inhomogeneous growth and toxin production had been noted in large heterogeneous foods before (69, 70).

The complex landscape of the BoNTs with eight serotypes, >40 subtypes, and seven different toxin producing species, combined with a heterogeneous distribution in foods and a short diagnostic time window challenges the laboratory diagnostics. Thus, parallel analysis of different clinical samples and suspected food items for the presence of BoNT and BoNT-producing clostridia is strongly advisable to deliver a sound and timely diagnosis. For non-homogeneous foods special care has to be taken for the selection of representative subsamples (e.g., meat, intestines or roe). Awareness of the diagnostic limitations of individual samples combined with early clinical suspicion of botulism can speed up laboratory confirmation, which can be clearly illustrated during this outbreak. Initially, for cases 1 and 2, it took 12 and 9 days, respectively, between hospitalization and arrival of a suitable clinical samples at the CL-NTC (Figure 1). In contrast, samples arrived at the CL-NTC in 2–5 days post hospitalization at the end of the outbreak (cases 6 and 7). This substantially shorter lag phase between hospitalization and sample dispatch might well be attributed to an increased awareness for foodborne botulism induced by fish products at the level of the clinicians and local health authorities.

In summary, this first international foodborne botulism outbreak due to a commercial product within the EU highlights not only the enduring risk associated with products consumed unheated but also the difficulties encountered in product traceability and recall, as well as in identification and prevention of factors leading to the occurrence and distribution of unsafe products. The complex international food distribution chains combined with the introduction of otherwise uncommon food products due to increasing migration calls for constant vigilance combined with continuing training of clinicians and health authorities in the recognition and management of rare diseases to further enhance timely recognition of unsafe products and as a consequence to enhance consumers' safety. This cross-national outbreak highlights the interplay of epidemiological and laboratory investigations combined with food traceability and consumer safety aspects to address and manage foodborne outbreaks, but moreover stretches that such an interdisciplinary interplay needs to be fostered, strengthened and at best well-orchestrated as one-health-approach at an international level to identify and handle future events.

## Data availability statement

The datasets presented in this study can be found in online repositories. The names of the repository/repositories and accession number(s) can be found in the article/supplementary material.

## Ethics statement

The animal study was reviewed and approved by Landesamt für Gesundheit und Soziales—Berlin.

## Author contributions

Wrote the manuscript: DH, CW-W, MC, CV, CF, BD, and MD. Performed laboratory or epidemiological investigations: DH, K-HJ, MC, PH, SM, JP, PT-D, KS, FB, KK, AJ, ST, CV, RC, MP, MN, MZ, AS, SV, LS, SW, BD, and MD. All authors reviewed and approved the final manuscript.
